# A Practical Treatise on the Diseases of Children

**Published:** 1853-11

**Authors:** 


### EDITORIAL.

*A Practical Treatise on the Diseases of Children*, by J FORSYTH MEIGS, M. D., &c. Second Edition, revised and enlarged. Philadelphia: Lindsay and Blakiston. 1853.

ONE characteristic of the present age is the rapid diffusion of knowledge. New facts and new principles, instead of being hoarded up as the property of a single observer, are scattered broad-cast over our land, to be appropriated by all who choose to read.

The frequent demand for new editions of standard works affords to their authors the opportunity of modifying their views as affected by their own experience, and of incorporating into their books the facts and observations of others.

Dr. Meigs has enhanced the value of the new edition of his work by an introductory essay on the clinical examination of children, by the re-arrangement and re-writing of the articles on croup, bronchitis, and pneumonia; by a discussion of the value of tracheotomy in croup; by the insertion of an article on atelectasis or imperfect expansion of the lungs; by a revision and partial re-writing of the article on scarlet fever, and by the addition of more than a hundred pages of new matter on diseases of the skin.

The diagnosis between spasmodic croup and membranous croup is always attended with more or less difficulty. The following comparison of symptoms, as given by Dr. Meigs, appears to us to be valuable:

Mild cases of spasmodic croup may be distinguished from membranous croup by a comparison of the different symptoms as they arise. The most important of these are: the invasion, in one sudden and almost invariably in the evening or night, in the other, slow and creeping, the paroxysm occurring indifferently day or night; the cough, in one hoarse and boisterous, in the other, hoarse and frequent at first, but rare and smothered towards the end; the voice, in one hoarse, but never scarcely whispering, and if so, only during the height, in the other hoarse at first, and soon permanently whispering or entirely lost; the cry, in one hoarse and stridulous only at the moment of the paroxysm, in the other permanently so; the respiration, in one stridulous and difficult only during the paroxysm, and in the interval perfectly natural, in the other, at first natural, becoming by degrees permanently stridulous, and attended by the most violent dyspnœa, with remarkable prolongation of the expiration; the fever, in one very slight and generally observed only during the nocturnal paroxysm, in the other much more considerable and permanent; and lastly, the duration, in one seldom more than two or three days, in the other rarely less than six, and very often eight or ten days. M. Trousseau states that the *hoarse-sounding*, *croupal* cough, is not a sign of the presence of exudation in the larynx, but rather of its absence; but, " when the cough, croupal at first, becomes less and less frequent, and ends with being nearly insonorous with suffocation, there is true croup, that is to say, with plastic exudation in the larnyx." This is precisely my own experience. The rare insonorous cough of M. Trousseau, is the condition which I have expressed by the term smothered.

In order to render the diagnosis still clearer, I add the following table, which is altered from one given by Rilliet and Barthez.

| MILD SPASMODIC LARYNGITIS. | PSEUDO-MEMBRANOUS LARYNGITIS. |
|---|---|
| Begins with coryza, and hoarse cough, or more frequently with a sudden attack of suffocation in the night. Fauces natural, or merely slight redness, as in simple angina. | In epidemic form, begins as pseudo-membranous angina. In sporadic form, invasion of slight hoarseness for a day or two.—There is fever, increase of the hoarseness, with hoarse, croupal cough; in half the cases pharyngeal exudation, and a little later paroxysms of suffocation. |
| After the paroxysm, the child seems well; the fever disappears, or is very slight. Voice natural or only slightly hoarse; not whispering. | The fever continues; stridulous respiration; prolonged and difficult expiration; cough hoarse and smothered; voice hoarse and whispering. |
| If the paroxysm returns, it is during the following night, and it is less severe; the hoarseness disappears; the cough becomes loose and catarrhal. | The dyspnœa and suffocation increase; the voice and cough are smothered or extinguished; stridulous respiration persists. |
| Duration seldom more than three days. | Duration seldom less than five or six. The hoarseness continues for several weeks. |
| Very rarely fatal. | Fatal in the majority of the cases. |

The only real difficulty in the diagnosis is the distinction between the grave form, and pseudo-membranous laryngitis or true croup unconnected with angina; and this, it would appear from all evidence, cannot in some cases be made with absolute certainty. The only certain and indubitable sign by which to distinguish them, is the presence of false membranes in the expectoration.—The existence of this symptom is proof positive of pseudo-membranous disease, but its absence is no proof that the case must be one of simple inflammation; for, even though the membrane has been exuded in large quantities within the larynx, it is not always thrown off by the effort of coughing or vomiting. To show the difficulty of the diagnosis, I will cite the case quoted by M. Valleix (*Loc. cit.* t. i. p. 211) from M. Hache, of a child supposed to be laboring under true croup, who was sent to the Children's Hospital in Paris in order to have the operation of tracheotomy performed. The absence of false membrane in the expectoration, and a slight remainder of clearness of the voice, occasioned the suspension of the operation. The child died, and no pseudo-membrane whatever was found in the larynx. The only lesions were moderate redness of the mucous membrane, without tumefaction, and without narrowing of the glottis; so that the fatal termination must be ascribed to spasmodic constriction of the glottis, or to tumefaction of that part which had disappeared after death.

Nevertheless, though the diagnosis is difficult, it can generally be made out with considerable certainty by attention to the following points. The pseudo-membranous form of the disease is often preceded or accompanied by the presence of false membranes in the fauces, which is not the case in spasmodic simple laryngitis; the symptoms of invasion of the former disease are less acute than those of the latter, the fever being less violent, and the restlessness and irritability less marked, than is usual in the simple affection, in which the general symptoms are severe from the first. The hoarseness of the voice and cough follow a different course in the two diseases; the progress of these symptoms being slow and gradual in the membranous, and much more rapid in the severe spasmodic form. The fever is violent throughout the attack in the severe spasmodic disease, whilst in the other form it seldom reaches a high degree of intensity. Lastly, the presence of portions of false membrane in the expectoration, in connection with the laryngeal symptoms, affords positive evidence of the existence of true croup.

Of the characters just enumerated as likely to aid us in distinguishing between severe spasmodic and true or membranous croup, I wish to call the reader's attention in greater detail to two, the first of which is the condition of the voice. This is, I have no doubt, much the most important single symptom. In membranous croup, the voice begins by being hoarse, but soon becomes weak, so that after the disease has lasted three or four days, it changes from hoarse to whispering; it becomes, in fact, suppressed. Now in severe spasmodic croup, the voice is hoarse at first, and becomes more so as the disease goes on, but it very rarely becomes whispering as in true croup, but almost always retains a good volume, so that when urged the child can speak out loudly. This is never the case in membranous disease, for, as the fibrinous exudation is deposited on the vocal cords and in the ventricles of the larynx, it suspends almost entirely the functions of those parts, and the voice is more or less completely suppressed. The remarks just made in regard to the voice, will apply also to the *cry*, which should be carefully studied in young infants.

Another symptom that ought to be closely scrutinized, is the stridor. This is, as might be expected, more marked in all its features, in true than in false croup, since in the former it depends on a permanent and considerable obstacle to the passage of air through the larynx, That tube is, in fact, completely coated over upon its internal superficies, with a more or less thick false membrane, which reduces materially its calibre, and impedes to a great extent, the passage of air, than does the mere inflammatory turgesence and swelling of the mucous membrane of the organ in severe spasmodic croup. On this account, therefore, the stridor in the respiration is louder, shriller, more persistent, more marked in the expiration, and attended with greater effort of the respiratory muscles to overcome the obstacle to the passage of the air in membranous than in severe spasmodic croup.

To conclude, there is in membranous croup, a slow, steady, and unrelenting progression of the symptoms, which is not observed in the spasmodic disease. From hour to hour, from day to day, we can perceive, so to speak, from the gradual and steady march of the disease, that a foreign body in the form of a fibrinous moulding, is being spread slowly over the cavity of the larynx. In severe spasmodic croup, on the contrary, the course of the symptoms is less regular; paroxysms of suffocation occur as in true croup, but when these are over, the child is often quite comfortable, the symptoms indicating a much less considerable permanent mechanical obstruction than in the other affection.

In reference to the treatment of membranous croup after mentioning emetics, of which he prefers alumn, (as recommended by Prof. Meigs), and mercurials, he says:

The application of astringent and caustic remedies to the fauces, is of great importance in all cases beginning simultaneously in the pharynx and larynx. In membranous angina, when the disease shows any disposition to extend into the larynx, the use of these remedies constitutes one of the most essential points in the treatment, but of this a full account will be given in the article on that disease. In all cases of true croup, however, whether attended with fibrinous deposit in the fauces or not, I believe it will be found useful to employ the nitrate of silver as a local application to the pharynx, and low down over the chink of the larynx. I have made use of solutions of different strength, from five to forty grains to the ounce, and prefer, on the whole, one from ten to twenty grains. This should be applied low down in the pharynx, so as to touch the epiglottis, by means of a mop made of a small piece of soft sponge fastened upon a bent whalebone. The application is to be repeated twice or three times a day, or more frequently, according to the violence of the symptoms, and the effects of the remedy.

The subject of tracheotomy is discussed at length. In reference to it, our author says:

In a consideration of the propriety of this operation in croup, there are, it seems to me, two points in particular to be examined: 1. Whether it offers any chance of success whatever; and 2, Whether it is in itself dangerous. Now, in regard to the first point, we find, on consulting the English medical authors, and they are almost universally of opinion that it is scarcely ever successful, and therefore unjustifiable. When we turn to the French writers, on the other hand, we find that most of the recent authoritative writers on the diseases of children and on practice, recommend it as a legitimate method of treatment, and even press it upon us, as offering much additional chance of safety to the patient. The success of the operation among the French has been as follows: M. Bretonneau had six recoveries in twenty operations; M. Leclerc, of Tours, had one in two; M. Velpeau, two in ten; and M. Petel, three in six (*Rilliet et Barthez*, *Mal. des Enfants*, t. i. p. 379). M. Valliex (*Loc. cit.* p. 389) states that he found, upon examining into the results of tracheotomy, that the operation had been successful in nearly *one out of three.* M. Trosseau, who has performed the operation so much more frequently than any one else, is said to have had one hundred and thirty-five operations of which only one in four was successful, up to the year 1850 (*L'Abeille M dicale*, June 1st, 1852, p. 146). Since 1850 his success has been much greater, owing to certain modifications in the canula he employes, and in the after-treatment of the case. In 1850, he had six operations, and in the first months of 1851, eleven. Of the seventeen seven were successful.

The same modifications have been introduced into the Children's Hospital at Paris, and with the following results. In the space of fifteen years, more than forty operations had been performed without a single cure. In 1850, after the changes just referred to had been made, nineteen operations were performed, of which six were successful, and in the first seven months of 1851, sixteen were performed, of which seven ended favorably (*L'Abeille Med*,, loc. cit.) Of the modifications made by M. Trousseau, in his methods, I shall speak hereafter.

The modifications of M. Trousseau alluded to are the following:

In the first place, the patients, owing to a general change in the mode of treatment of croup, are in a better condition for the operation than formerly. The treatment consists usually of cauterization of the fauces with a very strong solution of the nitrate of silver (one part to three of water) of insufflations of alum into the throat, and emetics. Bleeding and blisters are generally avoided, as both are thought to be injurious, the former by debilitating the patient, and the latter because they are painful, useless, and because they became covered with false membranes, which have sometimes caused the death of the patient, when the operation has been otherwise perfectly successful.

In the second place, M. Trousseau has abandoned the use of the caustic instillations into the larynx and trachea, which he formerly employed in every case. He rarely makes use of emollient injections. He now employs only a double canula instead of a single one as formerly, which latter he was obliged to remove two or three times in the twenty-four hours, a proceeding always painful and nearly always difficult during the first two days. Now, the parents remove the internal canula every few hours as often as it becomes clogged, and clean and replace it without causing pain or irritation. Formerly, the neck being left exposed to the air, the mucus in the canula and trachea dried up, and he resorted to frequent instillations of water, and a process of swabbing out with a sponge-mop, in order to remove these obstructions. Now, as soon as the operation is finished, he envelopes the neck in a cravat, so that the expired air is in part inhaled again, thus preserving its warmth and particularly its moisture. By this proceeding the drying up of the mucus in the trachea and bronchia is avoided, expectoration is much easier, and injections and swabbing are no longer necessary. M. Trousseau formerly left the wound exposed to the air, contenting himself with dressing it sometimes with a little charpie covered with cerate. The wound became covered with false membranes, inflamed violently, and sometimes became even gangrenous. Now, he places over the charpie a piece of waxed silk, pierced with a hole for the passage of the canula, by which means the incision is protected doubly by the cravat and by the shield of waxed silk. On the day after the operation, he cauterizes vigorously all the divided parts which become covered with false membranes, and repeats the cauterization two or three times until the surface of the wound is perfectly clean.

It would afford us pleasure to follow our author further had we space. The chapters on diseases of the skin are interesting although necessarily somewhat imperfect.

Dr. Meigs has done essential service to the profession in the publication of this work. We regard the improvements in the present edition as valuable, and can cordially recommend it to our readers.

For sale by D. B. Cooke & Co., 135 Lake st., Chicago. J.

